# Entedoninae wasps (Hymenoptera, Chalcidoidea, Eulophidae) associated with ants (Hymenoptera, Formicidae) in tropical America, with new species and notes on their biology

**DOI:** 10.3897/zookeys.134.1653

**Published:** 2011-10-06

**Authors:** Christer Hansson, Jean-Paul Lachaud, Gabriela Pérez-Lachaud

**Affiliations:** 1 Scientific Associate of Entomology Department, the Natural History Museum, London SW7 5BD, United Kingdom; 2 El Colegio de la Frontera Sur, Entomología Tropical, Av. Centenario Km 5.5, Chetumal 77014, Quintana Roo, Mexico; 3 Centre de Recherches sur la Cognition Animale, CNRS-UMR 5169, Université de Toulouse UPS, 118 route de Narbonne, 31062 Toulouse Cedex 09, France

**Keywords:** *Horismenus*, *Microdonophagus*, *Camponotus*, *Microdon*, ant parasitism, myrmecophile, taxonomy

## Abstract

Three new species of Eulophidae associated, or presumed to be associated with ants are described: two species of *Horismenus* Walker and one species of *Microdonophagus* Schauff. Information on the biology is also included. The two *Horismenus* species are from Chiapas, Mexico. *Horismenus myrmecophagus* **sp. n.** is known only from females and is a gregarious endoparasitoid in larvae of the weaver ant *Camponotus* sp. ca. *textor*. The parasitoids pupate inside the host larva, and an average of 6.7 individuals develops per host. This is the second time a species of genus *Horismenus* is found parasitizing the brood of a formicine ant of genus *Camponotus*. *Horismenus microdonophagus* **sp. n.** is described from both males and females, and is a gregarious endoparasitoid attacking the larvae of *Microdon* sp. (Diptera: Syrphidae), a predator on ant brood found in nests of *Camponotus* sp. ca. *textor*. The new species of *Microdonophagus*, *Microdonophagus tertius*, is from Costa Rica, and known only from the female. Nothing is known about its biology but since another species in same genus, *Microdonophagus woodleyi* Schauff, is associated with ants through its host, *Microdon* larva (with same biology as *Horismenus microdonophagus*), it is possible that also *Microdonophagus tertius* has this association. A new distributional record for *Microdonophagus woodleyi* is also reported, extending its distribution from Panama and Colombia to Brazil.

## Introduction

Natural enemies of ants include dipteran, strepsipteran and hymenopteran parasitoids (for a review see Wilson (1971), Kistner (1982), Hölldobler and Wilson (1990)). Species of several families of parasitic wasps have been reported parasitizing brood or adult ants, the Eucharitidae being the only monophyletic group, at the family level, where all members are parasitoids of ants (Heraty 2002). In contrast to eucharitids, parasitization of ants by eulophids is a rare event.

Four associations involving a eulophid wasp and an ant host have been reported to date, and all are genera belonging to the subfamily Entedoninae: an unidentified gregarious parasitoid, apparently closely related to the genus *Paracrias* Ashmead (identified by Gahan), was recorded parasitizing larvae of the myrmicine *Crematogaster acuta* (Fabr.) in Guyana (Wheeler and Wheeler 1924), the prepupae of another, unidentified species of *Crematogaster* were parasitized by *Myrmokata diparoides* Bouček (Bouček 1972) in Cameroon, *Pediobius marjoriae* Kerrich was reared from cocoons of *Lepisiota* sp. (referred to as *Acantholepis* sp.) (Formicidae: Formicinae) in Uganda (Kerrich 1973), and *Horismenus floridensis* (Schauff & Bouček) (referred to as *Alachua floridensis*) was found parasitizing the pupae of *Camponotus atriceps* (F. Smith) (referred to as *Camponotus abdominalis* Fab.) and of *Camponotus floridanus* (Buckley) (Formicidae: Formicinae) in Florida (Schauff and Bouček 1987). Three other species of Eulophidae (the first two are Entedoninae, the third a Tetrastichinae) have been reported associated with ant nests but direct parasitism on the ant brood was not clearly established in any of these cases: *Myrmobomyia malayana* Gumovsky & Bouček with nests of an ant species of the genus *Dolichoderus* in Malaysia (Gumovsky and Bouček 2005), an unidentified species of *Horismenus* from the bivouac and refuse deposits of the army ant *Eciton burchellii* (Westwood) (Rettenmeyer et al. 2011), and an unidentified species of *Tetrastichus* from a nest of *Myrmecocystus mexicanus* Wesmael in USA (Wheeler and Wheeler 1986). Finally, two species of Entedoninae are indirectly associated with ants as they parasitize insects living in ant nests: Kerrich (1973) reported *Pediobius acraconae* Kerrich from the last instar larva of *Acracona remipedalis* Karsh (Lepidoptera: Pyralidae) living in a nest of *Crematogaster depressa* (Latreille) or *Camponotus africana* Mayr (Formicidae: Myrmicinae) in Nigeria, and *Microdonophagus woodleyi* Schauff parasitizes larvae of *Microdon* sp. (Diptera: Syrphidae), living in nests of *Technomyrmex fulvus* (Wheeler) (referred to as *Tapinoma fulvum*) (Formicidae: Dolichoderinae) (Schauff 1986).

Here we describe two species of *Horismenus*, one parasitizing the brood of the weaver ant *Camponotus* sp. ca. *textor*, and the other parasitizing a syrphid myrmecophile associated with this ant species. A new species of *Microdonophagus* Schauff presumed to be associated with ants is also described. A new distributional record for *Microdonophagus woodleyi* is provided.

## Methods

Specimens for this study were either reared (*Horismenus* species) or collected manually (*Microdonophagus*), killed in alcohol, and subsequently critical point dried and mounted on cards for further studies. Observations of the specimens were made through a stereomicroscope, Nikon^©^ SMZ 1500 with a halogen ring light as light source. The colour photos were taken with a DS-Fi1 camera mounted on the stereomicroscope and the light source for the photos was a dome light manufactured from a description on http://www.cdfa.ca.gov/. Each picture was made from several photos taken at different levels of focus, and merged using Helicon Focus^©^. Micrographs are from uncoated specimens analyzed in low vacuum, with a JEOL^©^ JSM 5600 LV scanning microscope.

### Morphological abbreviations and acronyms

Abbreviations for morphological terms: DE = shortest distance between eyes in frontal view; DO = diameter of median ocellus; HE = height of eye; HW = height of fore wing; LC = length of median carina on propodeum; LG = length of gaster; LM = length of marginal vein; LS = length of hind tibial spur; LT = length of hind tarsus; LW = length of fore wing, measured from base of marginal vein to apex of wing; MM = length of mesosoma; MS = malar space; OOL = distance between one posterior ocellus and eye; PM = length of postmarginal vein; POL = distance between posterior ocelli; POO = distance between posterior ocelli and occipital margin; ST = length of stigmal vein; WC = greatest width of median carina on propodeum; WH = width of head; WM = width of mouth; WT = width of thorax. For illustrations of the morphological terms see http://www.neotropicaleulophidae.com/.

Collection acronyms used are: BMNH = The Natural History Museum, London, England; CH = collection of Christer Hansson; ECO-CH-AR = Arthropod Collection El Colegio de la Frontera Sur-Chetumal, Mexico; USNM = the United States National Museum of Natural History, Washington, D.C., USA.

## Taxonomy

### Genus Horismenus Walker

There are 400 species described of the almost exclusively New World genus *Horismenus* Walker, 1843, mostly from the Neotropical region (Hansson 2009a), but only 13 have been reported from Mexico. Host records are available for 99 *Horismenus* species (Pérez and Bonet 1984, Bonet 2008, Hansson 2009a). The genus shows a wide host spectrum including lepidopteran and coleopteran leaf-miners, seed-eating bruchids and curculionids, coccids, mantispids, spider eggs, and both dipteran and hymenopteran parasitoids. Members of this genus also display a variety of life styles. Records include both primary and secondary parasitoids, solitary or gregarious species, specialist or generalist parasitoids, and as far as is known all are endoparasitoids and presumably koinobionts. The species may attack eggs, larvae, or pupae of their hosts.

#### Horismenus myrmecophagus sp. n.

urn:lsid:zoobank.org:act:CE88218A-4A94-4FD3-B87C-09C5442AA1AA

http://species-id.net/wiki/Horismenus_myrmecophagus

**Figure 1.**
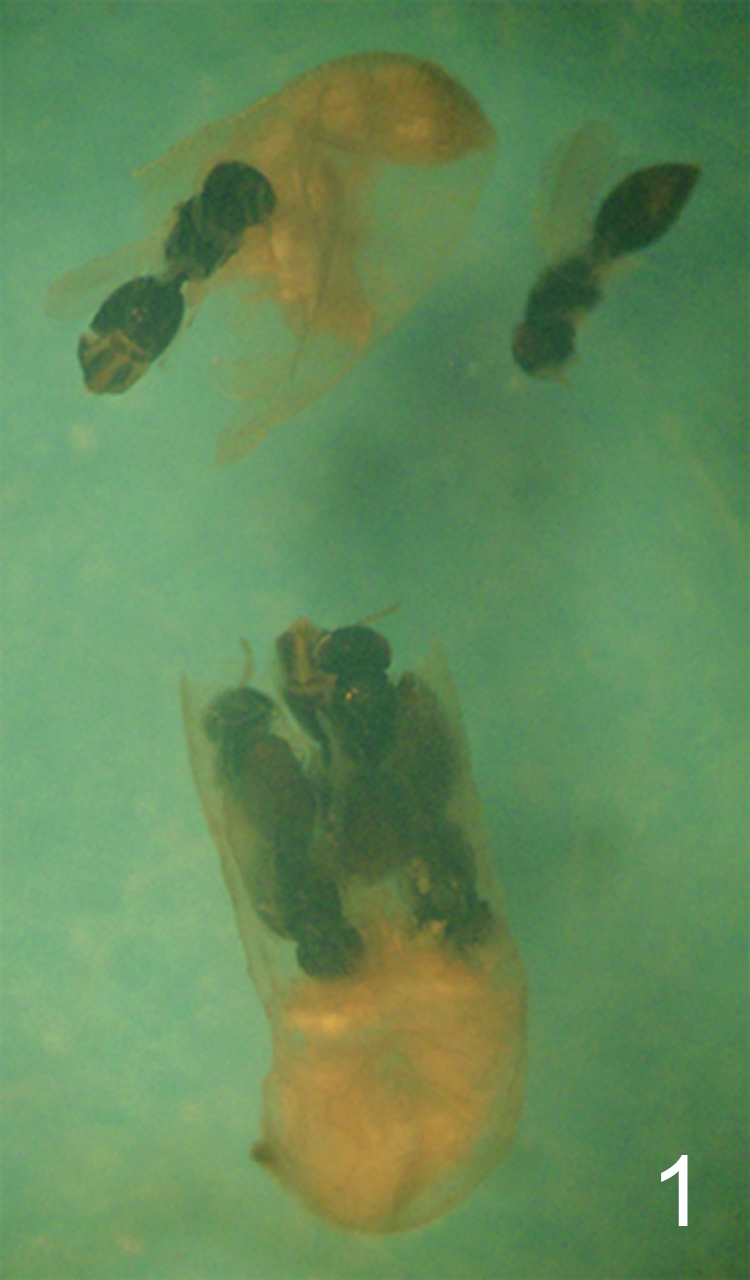
*Camponotus* sp. ca. *textor* larva parasitized by *Horismenus myrmecophagus*. *H. myrmecophagus* develops as a gregarious endoparasitoid. The ant larva has been cut open (its head is at the bottom of the picture). Several pupae of the eulophid parasitoid may be observed, some of them still inside the ant larva.

Figures 1 2–6 17, 21 23–24

##### Material.

HOLOTYPE female (BMNH), glued to a card, labelled “MEXICO: Chiapas, Tuxtla Chico, Rosario Izapa, 14°58'25"N, 92°09'19"W, 430 m, 25.ii.2010, G. Pérez-Lachaud & J.-P. Lachaud, reared from *Camponotus* sp. ca. *textor* pupa, nest no. 2, on mandarine *(Citrus reticulata*)”.PARATYPES. 1♀ with same label data as holotype (BMNH); 29♀ with same label and host data as holotype but collected from nest #3 28.ii.2010 (22♀ in BMNH, 2 ♀ in CH, 5♀ in ECO-CH-AR). Several paratypes have opaque and somewhat distorted wings due to premature killing in alcohol, i.e. before the wing membranes had hardened.

##### Diagnosis.

**Figures 2–6.**
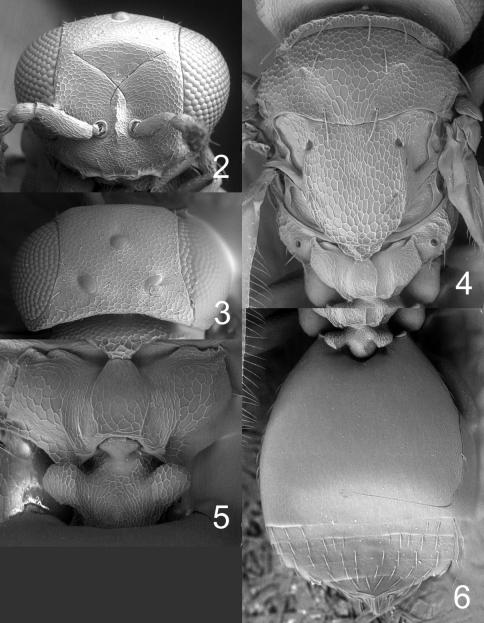
*Horismenus myrmecophagus* female: **2** head in frontal view **3** vertex **4** thoracic dorsum **5** propodeum **6** gaster in dorsal view.

 Frons with interscrobal area protruding and carinate (Fig. 2); scutellum entirely reticulate, without median groove and lateral mesh–rows (Fig. 4); fore wing speculum small and closed below (Fig. 21); all coxae white; propodeum with submedian grooves strongly reticulate and with anterolateral foveae weakly indicated anteriorly (Fig. 5); propodeal callus with five setae.

The species is very similar to *Horismenus alienus* Hansson, but differs mainly in the shape of the petiole which in *Horismenus alienus* has a strongly raised transverse carina dorsally, but *Horismenus myrmecophagus*has two strong and rounded projections dorsolaterally (Fig. 5); it differs also in sculpture of median propodeum: smooth in *Horismenus alienus*, but strongly reticulate in *Horismenus myrmecophagus*(Fig. 5).

##### Description.

*Female*. Length of body 1.1–1.4 mm. Scape white; pedicel and flagellum pale brown. Frons golden–green with purple tinges (Fig. 23). Vertex metallic bluish–green. Mesoscutum metallic bluish–green (Fig. 24). Scutellum dark golden–purple with green tinges (Fig. 24). Propodeum dark golden–purple (Fig. 24). Legs white. Wings hyaline. Petiole dark golden–purple. Gaster dark brown with metallic purple tinges.

Antenna as in Fig. 17. Frons (Fig. 2) with part just above frontal suture with raised and weak reticulation, remaining parts with raised and strong reticulation; frontal suture V–shaped, incomplete and not reaching eyes; antennal scrobes joining frontal suture separately. Vertex (Fig. 3) with raised and strong reticulation; without a median groove. Occipital margin rounded.

Mesoscutum with raised and strong reticulation (Fig. 4); notauli indistinct. Scutellum with raised and strong reticulation (Fig. 4), without median groove and lateral mesh–rows. Dorsellum slightly concave and with raised and strong reticulation. Propodeum with raised and strong reticulation (Fig. 5); propodeal callus with five setae. Coxae with raised and weak reticulation. Fore wing speculum small and closed below (Fig. 21); with 12 admarginal setae.

Gaster (Fig. 6) with first tergite with very weak reticulation posteriorly and laterally, otherwise smooth.

##### Ratios.

DE/DO 6.9; WH/DE 1.9; HE/MS/WM 2.4/1.0/2.0; POL/OOL/POO 2.5/1.0/1.1; WH/WT 1.2; LW/LM/HW 1.8/1.0/1.0; PM/ST 1.4; LC/WC 1.4; WG/WC 2.0; LS/LT 0.22; MM/LG 1.0.

*Male*. Unknown.

##### Etymology.

 Named after the feeding habits of the larva (from the Greek *myrmecophagus* = ant eater).

##### Distribution.

 Mexico (Chiapas).

##### Biology.

*Horismenus myrmecophagus*is a gregarious endoparasitoid of the larvae of *Camponotus* sp. ca. *textor*, a neotropical weaver ant. Parasitized host larvae spin a cocoon before their development is arrested, but no pupation occurs. Parasitized ant larvae are not modified in external form or color by the developing parasitoids, but changes in appearance were observed in the host at the end of the wasp larval development. In material preserved in alcohol, late instar larvae, pupae and teneral adults of the wasps can be readily observed inside ant larvae, within the host cocoon, but earlier developmental stages of the parasitoids could not be detected. The wasp larvae pupate inside the host larva. *Horismenus* individuals occupy almost the entire body of the host. Wasp pupae were found aligned on either part of the middle of the body of the host, their heads converging to the center, while the cephalic and caudal portions of the host larva were occupied by the host remains and the parasitoids meconia (Fig. 1). An average of 6.7 individuals developed per host (range: 4–12, mode: 7, n=27 parasitized cocoons examined). Adults emerge from the host cocoon through a unique, common hole pierced in the host larval cuticle and through the cocoon wall, but it is unknown whether adult wasps leave the nests to mate. Only females have been observed to date (all broods examined, where the sex of the parasitoid could be ascertained, were constituted by females (n=10 parasitized hosts)). The facts that only single sex broods parasitize any one host, and that only females are known, suggest that *Horismenus myrmecophagus* is a thelythokous species. Large ant larvae (presumably queens) have never been observed to be parasitized.

*Camponotus* sp. ca. *textor* (until now referred to in the literature as *Camponotus senex textor* Forel) is a common, dominant ant in shade coffee plantations in the Soconusco Region of Chiapas, Mexico (Philpott 2005). This species builds aerial nests on various native and introduced trees (*Inga* sp., *Citrus reticulata*, *Camponotus sinensis*) with the silk of their larvae. Nests measure up to 40 cm in diameter, and colonies may comprise up to 30.000 individuals (Pérez-Lachaud and Lachaud, unpublished data).

The host range of *Horismenus myrmecophagus* is unknown. It is possible that this species may attack other ant species occupying similar niches, given that certain species of *Horismenus* are known to be polyphagous (e.g. *Horismenus aeneicollis*, *Horismenus apantelivorus*, *Horismenus opsiphanis* or *Horismenus sardus*, see Hansson 2009a), and that other ants are known to be parasitized by eulophids in the type locality (e.g. *Pachycondyla crenata* (Roger), A. de la Mora personal comment), and in French Guiana (e.g. *Camponotus* (*Dendromyrmex*)sp., G. Pérez-Lachaud and J.-P. Lachaud, unpublished data), though their identity has not been confirmed yet.

##### Remarks.

 The similar species *Horismenus alienus* is known only from the female and its host/biology is unknown, but due to its morphological similarity to *Horismenus myrmecophagus*it is possible that *Horismenus alienus* is also a parasitoid of ants.

#### Horismenus microdonophagus sp. n.

urn:lsid:zoobank.org:act:1848E913-005D-48C1-8431-870D4FACB80F

http://species-id.net/wiki/Horismenus_microdonophagus

**Figures 7–11.**
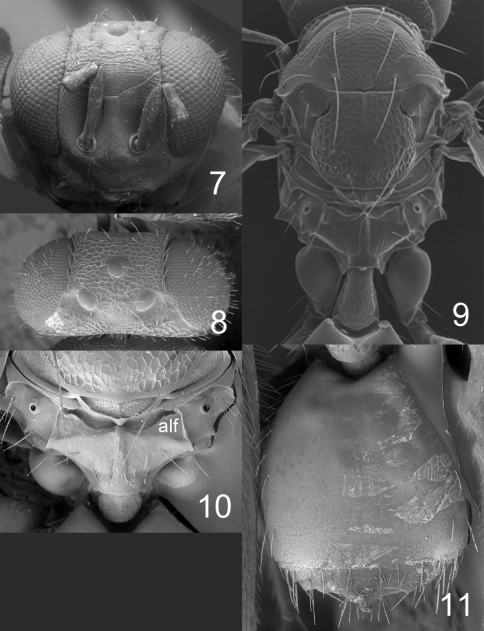
*Horismenus microdonophagus* female: **7** head in frontal view **8** vertex **9** thoracic dorsum and petiole **10** propodeum **11** gaster in dorsal view. Abbreviation alf = anterolateral fovea.

Figures 7–11 18–19, 22 25 27

##### Material.

 HOLOTYPE female (BMNH) glued to a card, labelled “MEXICO: Chiapas, Tuxtla, Chico, Rosario Izapa, 14°58'25"N, 92°09'19"W, 430 m, 28.ii.2010, G. Pérez-Lachaud & J.-P. Lachaud, reared from larva of *Microdon* sp., predator inside *Camponotus* sp. ca. *textor*, nest no. 3”. PARATYPES. 10 ♀ 2♂ with same label data as holotype (4 ♀ 1♂ in BMNH, 1♀ in CH, 5♀ 1♂ in ECO-CH-AR).

##### Diagnosis.

 Fore wing speculum covered with setae (Fig. 22); scutellum transverse, 0.75X as long as wide, entirely reticulate with raised and strong reticulation and with a narrow median groove in anterior half (Fig. 9); propodeum with a median carina but without submedian grooves (Fig. 10). This species is easy to recognize through these diagnostic features.

##### Description.

*Female*. Length 2.0 mm. Scape yellowish–brown, pedicel pale brown, flagellum dark brown. Frons dark golden–green (Fig. 25). Vertex golden–red. Mesoscutum golden–red with posterior 2/3 of midlobe metallic bluish–green (Fig. 26), to predominantly metallic bluish–green or golden–green. Scutellum golden with a median spot metallic bluish–green (Fig. 26), to predominantly metallic bluish–green. Propodeum metallic purple (Fig. 26). Coxae black to dark brown with golden–green tinges; femora, tibiae and tarsi yellowish–brown. Wings hyaline. Petiole black, shiny. Gaster metallic dark purple.

Antenna as in Fig. 18. Frons (Fig. 7) with interscrobal and clypeal areas and part just above frontal suture smooth, remaining parts with raised and strong reticulation with small meshes; frontal suture V–shaped, incomplete not reaching eyes; antennal scrobes join with frontal suture separately. Vertex (Fig. 8) with raised and strong reticulation, areas just behind posterior ocelli smooth; posterior part without median groove. Occipital margin rounded (Fig. 8).

Mesoscutum and scutellum with raised and strong reticulation (Fig. 9); notauli as indistinct impressions, forming posterior part of midlobe to an indistinct triangle. Dorsellum concave with raised and strong reticulation. Propodeum smooth (Fig. 10) or with raised and weak reticulation; median carina narrow and weak; propodeal callus with 5–7 setae and with 2–3 additional setae on median part of propodeum. Coxae smooth. Fore wing speculum absent or very small, obliterated by setae (Fig. 22); with 15 admarginal setae.

Gaster (Fig. 11) with first tergite smooth and shiny with a very weak reticulate band close to posterior margin.

##### Ratios.

 DE/DO 4.2; WH/DE 2.4; HE/MS/WM 2.7/1.0/1.4; POL/OOL/POO 3.1/1.0/1.6; WH/WT 0.9; LW/LM/HW 1.9/1.2/1.0; PM/ST 1.7; LC/WC 4.0; WG/WC 1.5; LS/LT 0.32; LP/WP 1.5; MM/LG 1.3–1.4.

*Male*. Length 1.6 mm. The male is similar to the female except: scape inflated (Fig. 19) and dark brown, slightly longer petiole and shorter gaster.

##### Ratios.

 HE/MS/WM 2.4/1.0/1.2; LP/WP 1.6; MM/LG 1.6.

##### Etymology.

 Named after the feeding habits of larvae (from the Greek *microdonophagus* = eater of *Microdon*).

##### Distribution.

 Mexico (Chiapas).

##### Biology.

*Horismenus microdonophagus* is a gregarious endoparasitoid of *Microdon* larvae (Diptera: Syrphidae), a predator on the brood of *Camponotus* sp. ca. *textor*. One *Microdon* sp. larva that was about to pupate was found inside a *Camponotus* nest. From this single host 79 females and 6 males of *Horismenus microdonophagus* emerged.

##### Remarks.

 One of the two males has the flagellum of both antennae missing, as have also some of the female paratypes, and the other male has the entire right flagellum and apical two flagellomeres of the left antenna missing. Only specimens in fair condition were included in the description, i.e. are type material. The remaining specimens were too fragmented to be included.

### Genus Microdonophagus Schauff

This is an exclusively Neotropical genus recorded from Brazil, Colombia, Costa Rica and Panama (Schauff 1986, Hansson 2002, 2009b). It now includes three species but only in one species, *Microdonophagus woodleyi*, is the biology known. This species is a gregarious endoparasitoid in larvae of *Microdon* sp. (Diptera: Syrphidae) living in nests of *Technomyrmex fulvum* (Formicidae: Dolichoderinae) (Schauff 1986). The same species also shows distinct sexual dimorphism, female being “normal” but male having several derived characters such as small eyes, reduced wings, and strongly inflated femora (Schauff 1986). Males are not known for the other two species.

#### Microdonophagus tertius

Hansson sp. n.

urn:lsid:zoobank.org:act:5DB16050-0C5D-41AE-A2EA-6D16981D2E2C

http://species-id.net/wiki/Microdonophagus_tertius

**Figures 12–16.**
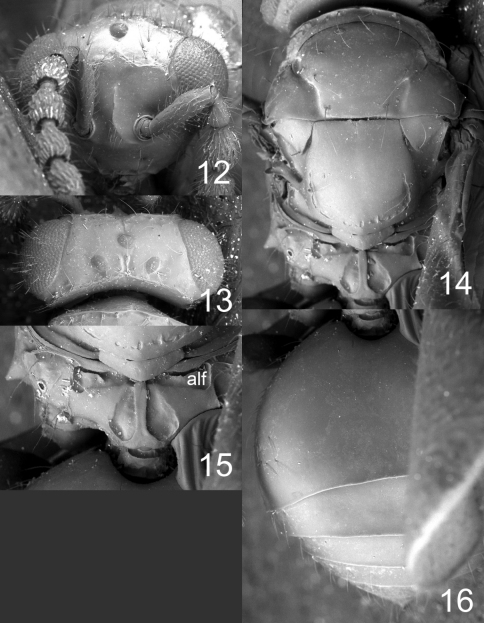
*Microdonophagus tertius* female: **12** head in frontal view **13** vertex **14** thoracic dorsum **15** propodeum **16** gaster in dorsal view. Abbreviation alf = anterolateral fovea.

Figures 12–16, 20, 28

##### Material.

 HOLOTYPE female (BMNH) glued to a card, labelled “COSTA RICA, Puntarenas, Parque Nacional Corcovado, Mosokha, Quebrada Hedionda, 15.iii–15.iv.2003, Khanaki.”

##### Diagnosis.

 This species is similar to *Microdonophagus levis* Hansson (Hansson 2009b) in its smooth and shiny thoracic dorsum but differs from the latter in several characters: scutellum without median groove (Fig. 14); propodeum with a narrow median carina and with distinct anterolateral foveae (Fig. 15); lower mesepimeron enlarged (Fig. 28) but not as enlarged as in *Microdonophagus levis* (Fig. 29).

##### Description.

*Female*. Length 2.0 mm. Scape yellowish–brown, pedicel and flagellum pale brown. Head and body including gaster dark brown and shiny. Coxae pale brown; femora, tibiae and tarsi yellowish–brown. Wings hyaline.

Flagellum without anelli, with three funicular segments and a two-segmented clava (Fig. 20). Frons smooth and shiny (Fig. 12), without antennal scrobes and frontal suture, with a narrow and sharp process (an interantennal crest) between toruli. Vertex smooth and shiny (Fig. 13). Occipital margin sharp (Fig. 13). Eyes with scattered long hairs (longer than in *Microdonophagus levis*).

Mesoscutum smooth and shiny (Fig. 14); midlobe with two pairs of setae; notauli as distinct grooves throughout. Scutellum smooth and shiny (Fig. 14); with one pair of setae; with sublateral grooves in posterior half. Propodeum with a narrow median carina (Fig. 15); with wide sublateral grooves; with distinct anterolateral foveae; propodeal callus with about eight setae; propodeal surface smooth. Fore wing with four setae (right fore wing) and six setae (left fore wing) respectively on dorsal surface of submarginal vein; costal cell bare; speculum small and closed below; postmarginal vein not visible.

Petiole hidden behind inflated gaster but appears to be about as long as wide, dorsal surface with strong sculpture. Gaster circular; gastral tergites smooth (Fig. 16).

**Figures 17–22.**
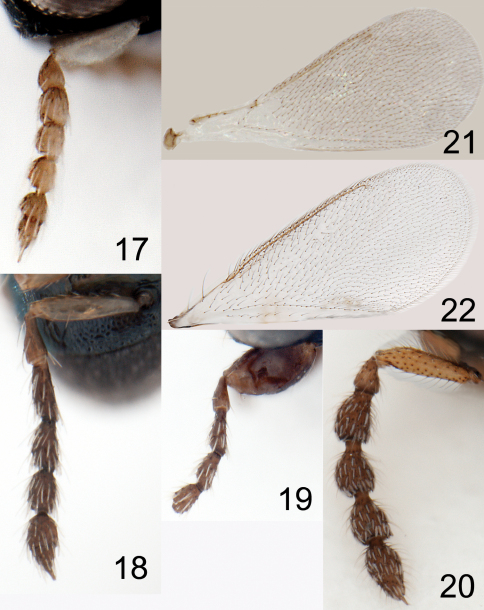
**17–20** antenna in lateral view: **17** *Horismenus myrmecophagus* female **18** *H. microdonophagus* female **19** *H. microdonophagus* male (apical 2 flagellomeres missing) **20** *Microdonophagus tertius* female. **21–22** right fore wing female: **21** *H. myrmecophagus* **22** *H. microdonophagus*.

**Figures 23–26.**
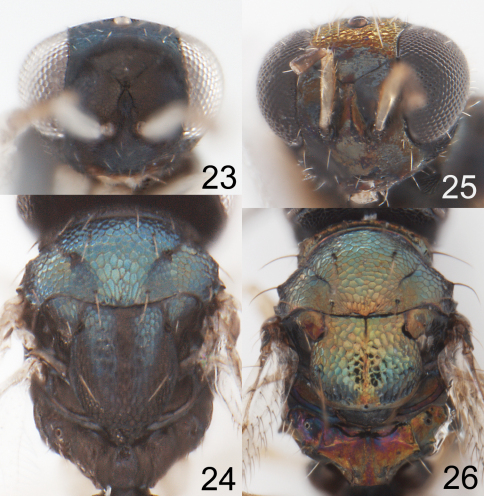
**23–24** *Horismenus myrmecophagus* female: **23** head in frontal view **24** thoracic dorsum. **25–26** *Horismenus microdonophagus* female: **25** head in frontal view **26** thoracic dorsum.

##### Ratios.

 HE/MS/WM 1.7/1.0/1.3; POL/OOL/POO 6.0/4.4/1.0; WH/WT 1.0; LW/LM/HW 1.7/1.0/1.0; LP/WP ca 1; MM/LG 1.0.

*Male*. Unknown.

##### Etymology.

 From the Latin *tertius* = third. Named for this being the third species described in the genus.

##### Distribution.

 Costa Rica.

##### Biology.

 Unknown, but possibly associated with ants, as is the type species of *Microdonophagus*, *Microdonophagus woodleyi*.

#### Microdonophagus woodleyi

Schauff

http://species-id.net/wiki/Microdonophagus_woodleyi

Figure 30

Microdonophagus woodleyi Schauff, 1986: 170–172. Holotype female in USNM.

##### Distribution.

 Brazil (new record, 1♀ from Santa Catarina, Nova Teutonia, 7.iv.1938, F. Plaumann, in BMNH), Colombia (Hansson 2002), Panama (Schauff 1986).

**Figures 27–31.**
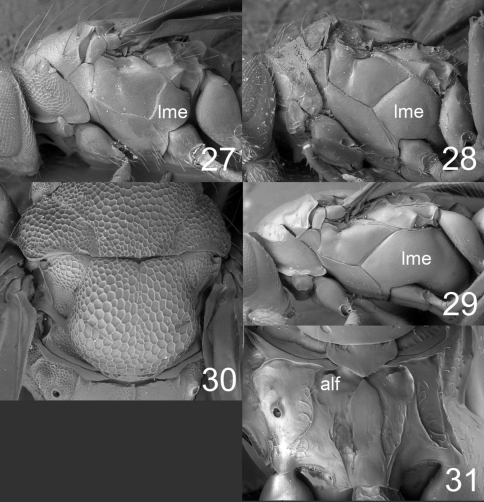
**27–29** mesosoma in lateral view females: **27** *Horismenus microdonophagus* **28** *Microdonophagus tertius* **29** *M. levis* Hansson **30** *M. woodleyi* Schauff, part of thoracic dorsum with scutellum. **31** *M. levis*, propodeum. Abbreviations alf = anterolateral fovea; lme = lower mesepimeron.

## Identification

*Horismenus* and *Microdonophagus* can be identified using the matrix key to Entedoninae genera on the website http://www.neotropicaleulophidae.com/. These two genera share certain characters that indicate that they are sister-groups: multiporous plate sensilla on flagellomeres with upper surface concave; propodeum with median carina, submedian grooves and anterolateral foveae. However, they also possess characters that show that each genus is a distinct monophyletic group:

**Table:** 

*Horismenus*	*Microdonophagus*
Antennal spicule with apical seta	Antennal spicule with apical peg (apomorphy)
Antennal scrobes as grooves	Antennal scrobes not visible (apomorphy)
Frontal suture as groove	Frontal suture not visible (apomorphy)
Axillar fovea present (apomorphy)	Axillar fovea missing
Lower mesepimeron normal	Lower mesepimeron enlarged (apomorphy)

### Species identification.

 (using modifications of the key in Hansson (2009a) below, but interactive keys on the website http://www.neotropicaleulophidae.com/, can also be used).

*Horismenus myrmecophagus*females run to subkey B and from there to couplet 5, first alternative, where *Horismenus myrmecophagus*is differentiated from *Horismenus alienus* as indicated above under diagnosis for *Horismenus myrmecophagus*.

*Horismenus microdonophagus* females run to subkey D and from there to couplet 8 where the second alternative is chosen and this leads to 9a instead of 9, and then:

**Table:** 

9a	Fore wing speculum small (Fig. 22) *and* propodeum with a complete median carina (Fig. 10)	*Horismenus microdonophagus* sp. n.
–	Fore wing speculum large, *or* median carina on propodeum mainly obliterated by reticulation	9

*Horismenus microdonophagus* males are difficult to include in the key because the antennal clava is missing. However, the combination of strongly inflated scape, transverse scutellum that is completely reticulate, small fore wing speculum, and propodeum without submedian grooves make males of this species easy to recognize (true also for females).

#### Key to all species of Microdonophagus

**Table:** 

1	Scutellum with raised and strong reticulation (Fig. 30)	*Horismenus woodleyi* Schauff (female)
–	Scutellum predominantly smooth and shiny (Fig. 14)	2
2	Lower mesepimeron (lme) strongly enlarged (Fig. 29); propodeum with indistinct anterolateral foveae (alf) (Fig. 31)	*Microdonophagus levis* Hansson
–	Lower mesepimeron (lme) smaller (Fig. 28); propodeum with distinct anterolateral foveae (alf) (Fig. 15)	*Microdonophagus tertius* sp. n.

## Discussion

Parasitization of ants is uncommon among Eulophidae and a survey of published literature showed that only four (possibly just three) eulophid genera, all Entedoninae, are recorded as parasitoids of ants. Apart from *Horismenus* (*Horismenus floridensis* (Schauff and Bouček 1987), and *Horismenus myrmecophagus*sp. n. (this article)), only an unidentified genus, possibly close to *Paracrias* (Wheeler and Wheeler 1924), the monotypic genus *Myrmokata* (Bouček 1972), and *Pediobius* (Kerrich 1973) have been undoubtedly associated as parasitoids of ants. The record in Wheeler and Wheeler (1924) is “apparently closely related to the genus *Paracrias* Ashmead, but I cannot identify it positively, even generically”, attempt for identification and remark by A.B. Gahan. *Paracrias* spp. apparently exclusively target the order Coleoptera (Hansson 2002, Pikart et al. 2011), so *Paracrias* seems an unlikely candidate as a parasitoid of ants. The concepts of the genera *Paracrias* and *Horismenus* – which have morphological features in common (Hansson 2002) – were unclear at the time (1924) of the identification, so possibly this record actually also concerns a *Horismenus* species. Parasitism of ants is suspected but not verified for *Myrmobomyia* (Gumovsky and Bouček 2005), also an entedonine Eulophidae.

Systematic surveys of macro- and microfauna biodiversity directly within ant colonies are lacking (Hugues et al. 2008), especially in the Neotropics, and the hosts of most species of described eulophids are unknown. Parasitization of *Camponotus* sp. ca. *textor* colonies by *Horismenus myrmecophagus* is very low (less than 1% of ant pupae, Pérez-Lachaud and Lachaud, unpublished data). The very low prevalence of these myrmecophiles within ant nests, and the absence of targeted sampling methods to access their full biodiversity, may account for the rarity of records of eulophids associated with ants. The fact that in this single study two species of eulophids were recorded associated with ants stresses the likelihood that other eulophid species may also be associated with ants.

One hypothesized evolutionary path to parasitism of nest-building Hymenoptera, e.g. ants, by Hymenoptera parasitoids is via their non-hymenopterous symbionts, and then at some point in time a host shift has occurred (Hanson et al. in Hanson and Gauld 1995), as possibly exemplified by the two *Horismenus* species described here. We know nothing about the phylogenetic relationship between these two species but it would be interesting to know if *Horismenus myrmecophagus* evolved after *Horismenus microdonophagus*, thus possibly supporting this hypothesis.

## Supplementary Material

XML Treatment for Horismenus myrmecophagus

XML Treatment for Horismenus microdonophagus

XML Treatment for Microdonophagus tertius

XML Treatment for Microdonophagus woodleyi
